# Low-dose vs. standard-dose alteplase for Chinese patients with acute ischemic stroke: A propensity score analysis

**DOI:** 10.3389/fneur.2023.1120547

**Published:** 2023-02-21

**Authors:** Jiawen Xu, Xi Chen, Yanan Xie, Yi Wang, Shidong Chen, Qiang Dong, Yi Dong, Kun Fang

**Affiliations:** Department of Neurology, Huashan Hospital, Fudan University, Shanghai, China

**Keywords:** ischemic stroke, thrombolysis, acute stroke therapy, recombinant tissue plasminogen activator, symptomatic intracranial hemorrhage

## Abstract

**Background and purpose:**

Previous studies have stimulated debates on low-dose alteplase administration in acute ischemic stroke (AIS) among the Asian population. We sought to evaluate the safety and efficacy of low-dose alteplase in Chinese patients with AIS using a real-world registry.

**Methods:**

We analyzed data from the Shanghai Stroke Service System. Patients receiving alteplase intravenous thrombolysis within 4.5 hours were included. These patients were divided into the low-dose alteplase group (0.55–0.65 mg/kg) and the standard-dose alteplase group (0.85–0.95 mg/kg). Baseline imbalances were adjusted by using the propensity score matching. The primary outcome was mortality or disability, which was defined as the modified Rankin scale (mRS) score ranging from 2 to 6 at discharge. The secondary outcomes were in-hospital mortality, symptomatic intracranial hemorrhage (sICH) and functional independence (mRS score 0–2).

**Results:**

From January 2019 to December 2020, a total of 1,334 patients were enrolled and 368 (27.6%) were treated with low-dose alteplase. The median age of the patients was 71 years, and 38.8% were female. Our study showed that the low-dose group had significantly higher rates of death or disability (adjusted odds ratio (aOR) = 1.49, 95% confidence interval (CI) [1.12, 1.98]) and less functional independence (aOR = 0.71, 95%CI [0.52, 0.97]) than the standard-dose group. There was no significant difference in sICH or in-hospital mortality between the standard-dose and low-dose alteplase groups.

**Conclusions:**

Low-dose alteplase was related to a poor functional outcome without lowering the risk of sICH, compared with standard-dose alteplase for AIS patients in China.

## 1. Introduction

Intravenous thrombolysis using alteplase (0.9 mg/kg) for acute ischemic stroke (AIS) within 4.5 hours of onset has been proven effective (1, 2). Despite the solid evidence on the efficacy of alteplase, a higher risk of symptomatic intracranial hemorrhage (sICH) after thrombolysis still cannot be ignored. Hence, some Japanese trials compared two alteplase dosages of low dose (0.6 mg/kg) and standard dose (0.9 mg/kg) in AIS patients and indicated that a low dose of alteplase might be more suitable for Asian people (3, 4). Furthermore, the low-dose alteplase treatment has been strongly recommended for Japanese AIS patients within 4.5 hours by Japan Stroke Society Guidelines (5).

However, some studies in China suggested that the standard dosage might have better clinical outcomes than the low-dose alteplase without increasing the incidence of sICH (6, 7). The ENhanced Control of Hypertension And Thrombolysis strokE study (ENCHANTED) trial, as the only randomized controlled trial (RCT), has reported a lower incidence of sICH in the low-dose alteplase group but not achieved non-inferiority of low-dose to standard-dose alteplase for death or disability at 90 days (8). In our previous pool-data analysis, the low dosage was still considerable based on several regional databases (9).

Due to the high cost of a new RCT and the narrow gap expected to be found between dosages, we tried to investigate whether low-dose alteplase can bring better safety and a comparable functional outcome than standard dosage in Chinese patients with AIS using our real-world database.

## 2. Materials and methods

### 2.1. Study population

All data of this study were obtained from the Shanghai Stroke Service System (4S) registry, which was designed as a regional stroke network tracking real-time data on stroke care performance in Shanghai metropolitan area. The protocol of the 4S network has been reported previously and this study was approved by Institutional Review Board in Huashan hospital, with the waiver of consent as no identifying information was collected (10).

At all stroke centers in Shanghai, patients 18 years or older diagnosed with ICD-10 stroke codes (I63, I61, and G45) at discharge were registered in the 4S database. The eligibility criteria included: (1) aged 18 years or older; (2) had a clinical diagnosis of ischemic stroke with ICD-10 codes I63 at discharge; (3) received thrombolysis treatment within 4.5 hours of symptom onset; (4) no definite indication nor contraindication for either dose of alteplase. Patients with incomplete medical records were excluded. Based on the dosage of alteplase they used, the eligible patients were divided into low-dose alteplase group (0.55–0.65 mg/kg) and standard-dose alteplase group (0.85–0.95 mg/kg).

### 2.2. Data collection

Each stroke center in Shanghai used the electronic medical record with the web-based collection tools to document the stroke care procedures and automatically extract all medical data (10). Data of eligible AIS patients receiving thrombolysis with alteplase were analyzed in our study.

### 2.3. Outcomes

The primary functional outcome was death or disability, defined as modified Rankin scale (mRS) score 2-6 at discharge. The secondary functional outcome included in-hospital mortality, sICH, functional independence (mRS score 0–2) and distribution of scores on the mRS (ordinal shift analysis). The definition of sICH was Heidelberg Bleeding Classification (11).

### 2.4. Data analysis

Continuous data were expressed as mean (standard deviation) or median (interquartile range), and categorical data were described as frequency and percentage. Wilcoxon rank-sum test was used for continuous outcomes. All categorical variables were tested by Pearson χ^2^ test or Fisher's exact test separately.

Propensity score matching (PSM) was used to adjust baseline imbalances. The propensity score was estimated with the multivariable logistic regression model, with the IV-alteplase dose (standard dosage and low dosage) as the dependent variable and all baseline characteristics in Table 1 as covariates. Patients with low-dose therapy were matched 1:4 to those with standard-dose therapy according to the propensity score, with replacement, using the nearest neighbor matching with a 0.2 caliper (propensity score-matched cohort). Bias reduction after PSM was evaluated using standardized mean differences in covariate means. We performed a sensitivity analysis to assess the robustness of the results with a 1:1 matched design.

**Table 1:** Baseline characteristics of the patients after propensity score matching.

	**Standard-dose alteplase group, No. (%)**	**Low-dose alteplase group, No. (%)**	***P*-value**
Patients, No.	966	368	
Age, y, median (IQR)	71 (63, 79)	75 (64, 86)	**< 0.001**
Men	1,775 (64.8)	268 (61.0)	0.44
**Medical history**			
Stroke	195 (20.2)	81 (22.0)	0.46
Diabetes	218 (22.6)	84 (22.8)	0.92
Hypertension	592 (61.3)	223 (60.6)	0.82
Dyslipidemia	30 (3.1)	10 (2.7)	0.71
Myocardial infarction	12 (1.2)	4 (1.1)	0.82
Atrial fibrillation	101 (10.5)	44 (12.0)	0.43
Smoking	302 (31.3)	102 (27.7)	0.21
**TOAST classification**			**< 0.001**
Large-artery atherosclerosis	502 (52.0)	205 (55.7)	
Cardioembolism	149 (15.4)	59 (16.0)	
Small-vessel occlusion	264 (27.3)	70 (19.0)	
Other/unknown	51 (5.3)	34 (9.2)	
Premorbid mRS score ≤ 3	883 (91.4)	332 (90.2)	0.50
NIHSS, median (IQR)	4 (2,9)	4 (2,10)	0.79
LDL, mg/dL, median (IQR)	2.8 (2.2, 3.4)	2.8 (2.2, 3.4)	0.95
Time from stroke onset to intravenous thrombolysis, hr, median (IQR)	2.6 (1.9, 3.5)	2.5 (1.8, 3.4)	0.54

IQR, interquartile range; SD, standard deviation; CI, confidence interval; LDL, low-density lipoprotein; NIHSS, National Institutes of Health Stroke Scale [range, 0–42 (most severe)]; mRS, modified Rankin scale [range, 0-6 (most severe)]; y, years; hr, hours. P ≤ 0.05 was shown in bold.

Logistic regression was used to compare the functional outcome between the standard- and low-dose alteplase groups. When comparing the outcomes, cumulative incidences with a 95% confidence interval (CI) were presented and adjusted by patient features, including age, sex, history of ischemic stroke, hypertension, diabetes, dyslipidemia, atrial fibrillation (AF), myocardial infarction, baseline National Institutes of Health Stroke Scale (NIHSS) score, time from onset to treatment, premorbid modified Rankin scale (mRS) score and TOAST classification. The functional outcomes in subgroups were divided based on demographic variables (sex, age ≤ 70 vs. >70 years), TOAST classification, clinical severity (baseline NIHSS scores ≤ 10 vs. > 10), time from onset to treatment (≤ 3 vs > 3 hours) and premorbid mRS (0–1 vs. 2–5). All tests were 2-sided and a *p*-value < 0.05 was considered statistically significant. Data analyses were conducted with Stata/SE 15.0.

## 3. Results

**Figure 1:**
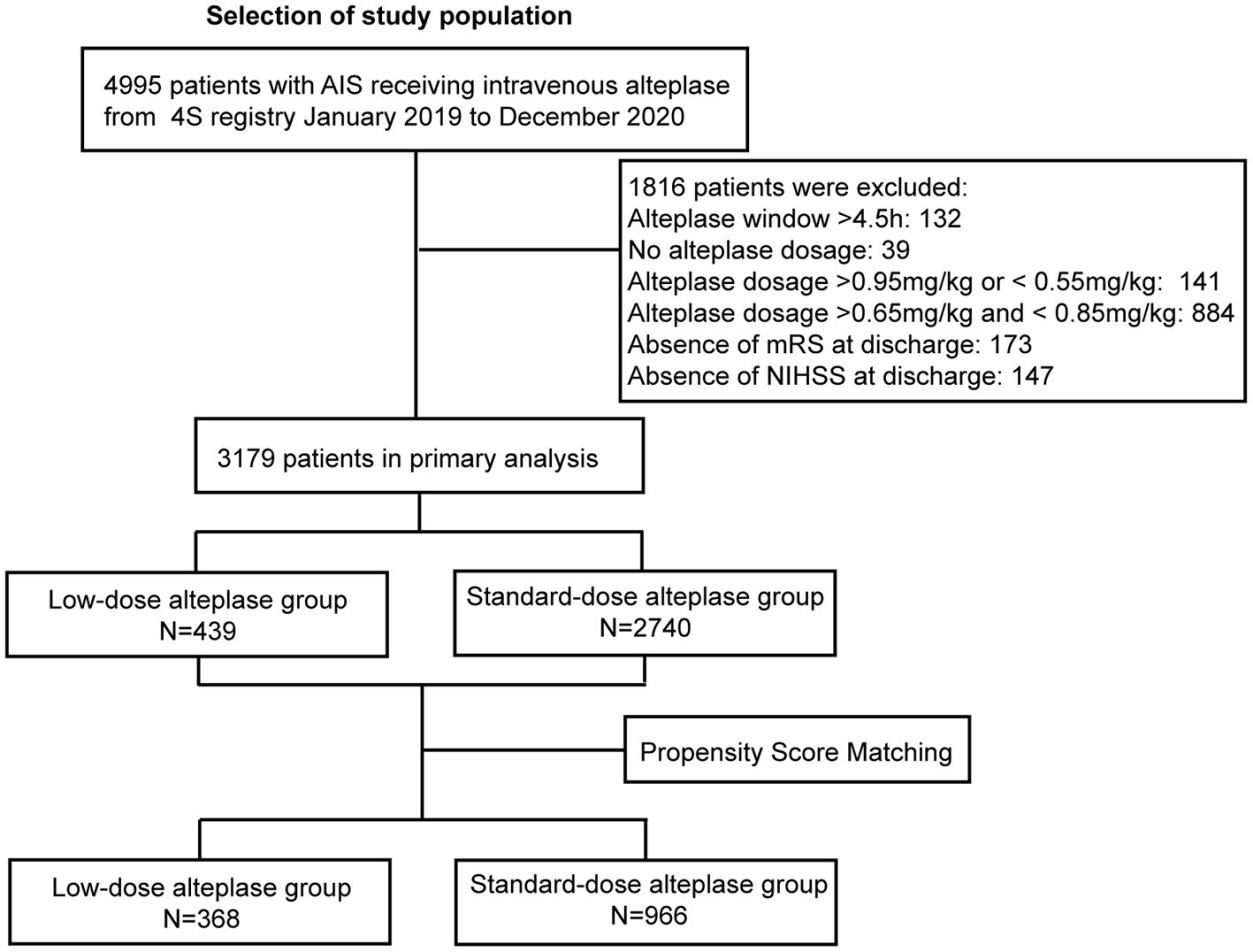
Study flow chart and numbers of eligible patients in each group. 4S, Shanghai Stroke Service System; AIS, acute ischemic stroke; NIHSS, National Institutes of Health Stroke Scale [range, 0–42 (most severe)]; mRS, modified Rankin scale [range, 0–6 (most severe)].

From Jan 2019 to Dec 2020, a total of 4995 patients received thrombolysis with alteplase. After excluding the cases with missing data (ie, the un-noted dosage of alteplase, treatment extended to the 4.5-hours window, absence of the mRS score or the NIHSS score at discharge), 3,179 patients were included in the study. The flowchart was shown in Figure 1. Baseline characteristics were shown in Supplementary Table 1. The patients in the low-dose group were older than those in the standard-dose group. Also, the low-dose group had a higher proportion of patients with a history of stroke, AF, or disability. After adjustment of PSM, 1,334 AIS patients were included in the final analysis. Among them, 368 (27.8%) patients received low-dose alteplase. The median age of the patients was 71 years, and 38.8% were female. The baseline characteristics in both groups after PSM were presented in Table 1. After PSM, the baseline characteristics were balanced except for age and TOAST classification; the absolute standardized differences were basically within an acceptable margin of 0.1 (Supplementary Table 2).

**Figure 2:**
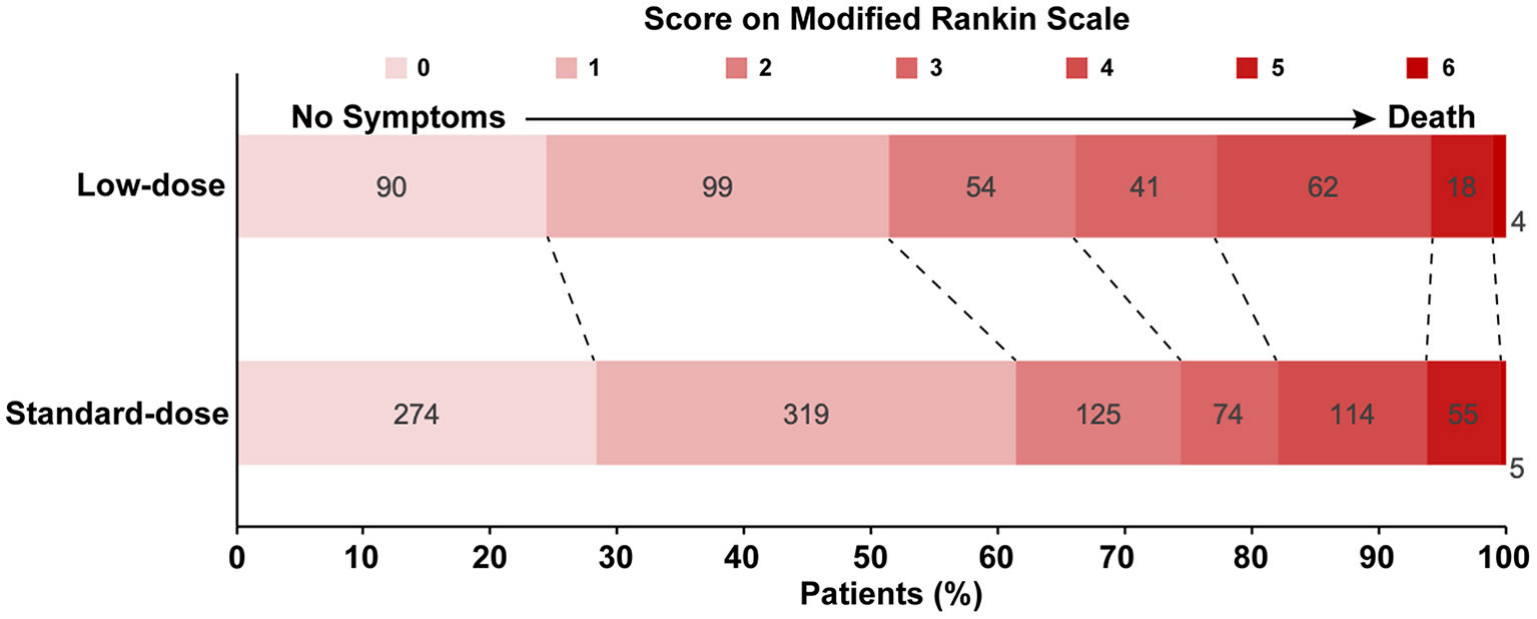
Distribution of Modified Rankin Scale Scores on discharge by low-dose vs. standard-dose alteplase in propensity score matching dataset.

The primary and secondary outcomes were summarized in Table 2. The low-dose group showed its association with a higher rate of mortality or disability (adjusted odds ratio (aOR) = 1.49, 95%CI [1.12, 1.98], *p* = 0.006) than the standard-dose group. Treatment with low-dose alteplase also resulted in a lower rate of mRS score 0-2 than that with standard-dose alteplase (aOR = 0.71, 95% CI [0.52, 0.97], *p* = 0.03). The results were similar to the univariate regression model. The distribution of mRS scores at discharge was shown in Figure 2. The common OR across the mRS scores between the low- and standard-dose alteplase was 1.36 (95% CI [1.10, 1.69]) in the univariate analysis, but without statistical significance after adjustment of baseline characteristics. In addition, no significant difference was found in the risk of in-hospital mortality or sICH for two groups. The sensitivity analysis for a 1:1 matched design showed generally similar results (Supplementary Table 3). Other serious adverse events during the hospitalization according to assigned treatment were presented in Table 3. There was a trend toward a higher incidence of pulmonary embolism in the low-dose alteplase group.

**Table 2:** Outcome according to assigned treatment in the propensity score matching dataset.

**Outcome**	**Standard-dose alteplase, No. (%)**	**Low-dose alteplase, No. (%)**	**Unadjusted OR (95% CI)**	***P*-value**	**Adjusted OR (95% CI)^†^**	***P*-value**
**Primary**						
mRS 2-6	373/966 (38.6)	179/368 (48.6)	1.51 (1.18, 1.92)	**0.001**	1.49 (1.12, 1.98)	**0.006**
**Secondary**						
sICH	30/966 (3.1)	8/368 (2.2)	0.69 (0.31, 1.53)	0.36	0.59 (0.25,1.37)	0.22
In-hospital mortality	5/966 (0.5)	4/368 (1.1)	2.11 (0.56, 7.91)	0.27	1.93 (0.47, 7.90)	0.36
mRS 0-2	718/966 (74.3)	243/368 (66.0)	0.67 (0.52, 0.87)	**0.003**	0.71 (0.52, 0.97)	**0.03**
mRS, median (IQR)	1 (0, 3)	1(1,3)	1.36 (1.10, 1.69)	**0.005**	1.21 (0.97, 1.51)	0.09

The propensity score was derived from the model with sex, age, history of ischemic stroke, hypertension disease, diabetes, dyslipidemia, atrial fibrillation, myocardial infarction, baseline NIHSS score, premorbid modified Rankin Scale, time from onset to treatment, baseline LDL-C and TOAST classification. IQR, interquartile range; NIHSS, National Institutes of Health Stroke Scale [range, 0–42 (most severe)]; mRS, modified Rankin scale [range, 0–6 (most severe)]; OR, odds ratio.^†^Adjusted for age, sex, history of ischemic stroke, hypertension disease, diabetes, dyslipidemia, atrial fibrillation, myocardial infarction, baseline NIHSS, time from onset to treatment, premorbid mRS and TOAST classification. P < 0.05 was shown in bold.

**Table 3:** Other serious events during hospitalization according to assigned treatment.

**Variable**	**Standard-dose alteplase group, No. (%)**	**Low-dose alteplase group, No. (%)**	***P*-value**
Recurrent stroke	44 (4.6)	16 (4.4)	0.87
Cardiac ischemia	5 (0.5)	2 (0.6)	1.00
Gastrointestinal Bleeding	14 (1.5)	2 (0.6)	0.26
Pulmonary embolism	6 (0.6)	7 (1.9)	0.06
Seizure	11 (1.1)	1 (0.3)	0.20
Hydrocephalus	1 (0.1)	0 (0)	1.0
Deep venous thrombosis	8 (0.8)	1 (0.3)	0.46

**Figure 3:**
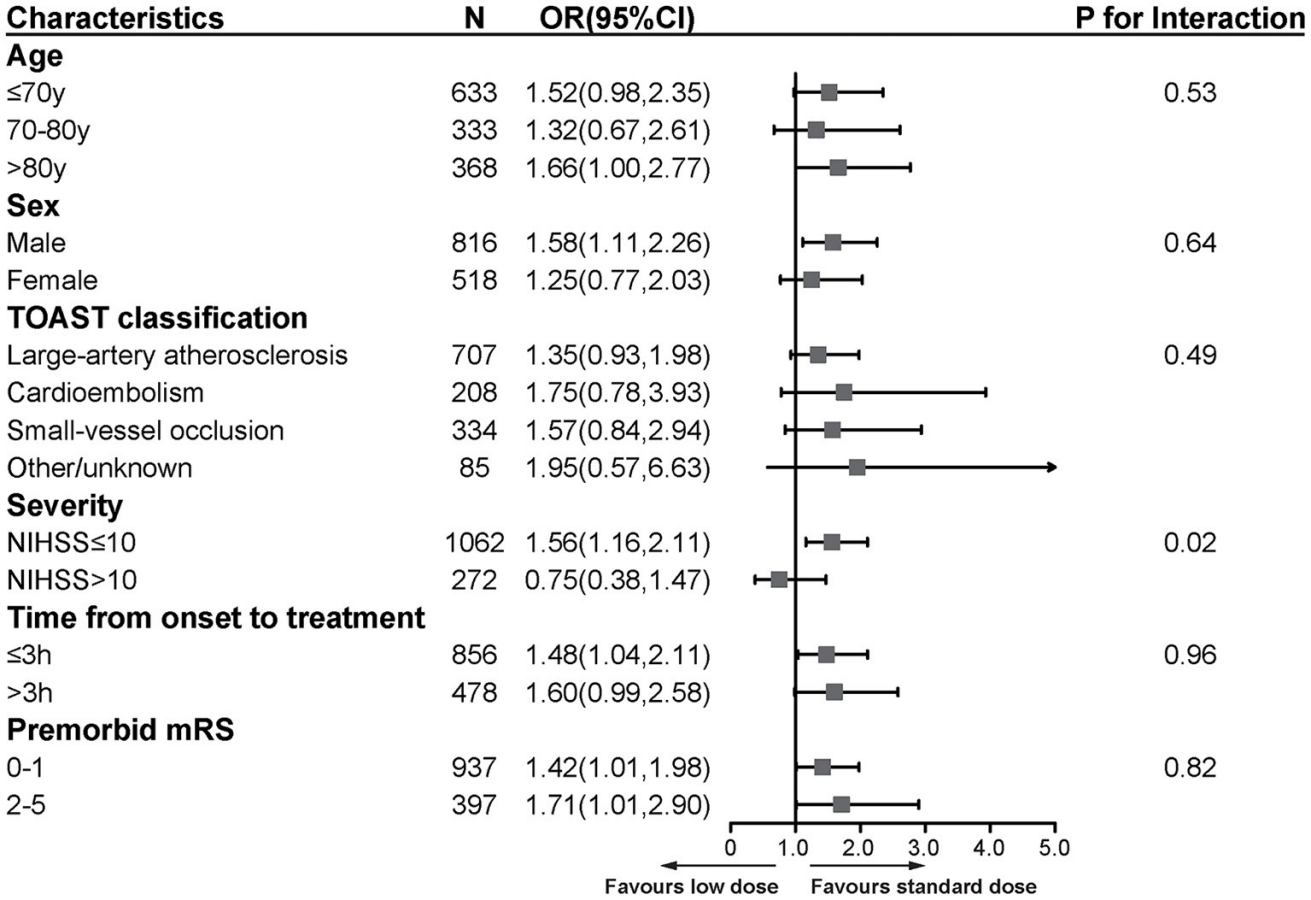
Subgroup analyses of primary functional outcome between low-dose and standard-dose group in propensity score matching dataset. The forest plot shows the difference in the primary functional outcome across all subgroups. The primary functional outcome was death or disability at discharge, defined by scores of 2 to 6 on the mRS [range, 0 (no symptoms) to 6 (death)]. The OR was calculated by using logistic regression, taking the following variables into account: age, sex, medical history (ischemic stroke, hypertension disease, diabetes, dyslipidemia, atrial fibrillation, myocardial infarction), baseline NIHSS, time from onset to treatment, premorbid score and TOAST classification. NIHSS, National Institutes of Health Stroke Scale [range, 0–42 (most severe)]; mRS, modified Rankin scale [range, 0–6 (most severe)]; OR, odds ratio.

We performed subgroup analyses by age, gender, stroke subtypes, stroke severity, time from onset to treatment, and premorbid mRS score (Figure 3). All characteristics did not modify the treatment effect, except for stroke severity (*p* = 0.02). The patients with baseline NIHSS score ≤ 10 benefited more from the standard-dose alteplase therapy (OR = 1.56, 95%CI [1.16, 2.11], *p* = 0.003).

## 4. Discussion

Our study demonstrated that patients receiving low-dose alteplase were associated with a higher risk of death or disability than those receiving standard-dose alteplase. Moreover, patients treated with low-dose alteplase were not associated with a lower sICH rate, and tend to have a higher rate of pulmonary embolism. Mild and moderate stroke patients with a baseline NIHSS score ≤ 10 may have better outcomes when treated with standard-dose alteplase.

Despite that a dose of 0.9 mg/kg alteplase is widely recommended by most guidelines for AIS patients (12–14), the high risks of sICH after thrombolysis with alteplase cannot be ignored, particularly among Asians (15). The J-ACT trial showed that the rate of mRS 0-1 at 90 days and the incidence of sICH in Japanese AIS patients receiving 0.6 mg/kg alteplase were comparable to 0.9 mg/kg alteplase treatment of the NINDS trial (1, 4). The results of several non-randomized trials in Japan subsequently showed the similar efficacy and safety outcome of low dosage thrombolysis with alteplase when compared with the standard dosage (3, 16, 17). In 2016, the ENCHANTED trial, as the only RCT involving 3310 AIS patients (63% Asian), aimed to compare low-dose with standard-dose alteplase. Although the trial failed to achieve the non-inferiority of low-dose as compared to standard-dose alteplase concerning the outcome of death or disability at 90 days (8), it also did not prove the superiority of standard-dose alteplase. There were significantly fewer sICH and trends toward the lower mortality rate with low-dose alteplase. Therefore, low-dose alteplase performed well in the safety outcome, which provided an alternative approach for both doctors and patients to make the personalized decision for the individual patient with AIS.

Compared with the ENCHANTED trial, our study achieved a similar efficacy outcome. What is new in our study, clinical physicians were more likely to administrate low dosage due to elder age and combined with AF. Our study found no association between alteplase dosage and the rate of sICH or mortality, which was different from the ENCHANTED trial. However, our findings were consistent with the results of TIMS-China (6, 7). An Asian stroke registry study also found no association between alteplase dosage and sICH risk (9). Our results might be the reflection of the real-world or false-negative error, attributed to the confounding bias of the observational study.

In subgroup analyses, we found that patients with mild-moderate stroke (NIHSS ≤ 10) in the standard-dose group were more likely to show a favorable outcome. However, the significant heterogeneity of stroke severity might be caused by a small sample of patients with a baseline NIHSS > 10. Our previous study showed that in mild stroke patients (NIHSS ≤ 4), there was no difference in the sICH risk and clinical improvement between both dosages (18). Therefore, more studies on the effectiveness and safety of low-dose thrombolysis for patients with different stroke severity are still needed.

The ENCHANTED trial raised the concern of the high risk of sICH in standard-dose alteplase treatment. Considering the bleeding-prone constitution of the Asian population, a secondary analysis of the ENCHANTED trial showed similar efficacy and safety outcomes of both doses of alteplase in both Asians and non-Asians (19). Also, the clinical use of low-dose alteplase in AIS patients with older age and other high-bleeding risks remained uncertain. Our subgroup study suggested that low-dose alteplase might be related to unfavorable functional outcomes even in patients older than 80.

Our study had several limitations. Firstly, our research population was limited to the Shanghai metropolitan. The management of risk factors and other demographic characteristics may not extend to that of other regions in China. Secondly, it was an observational retrospective study based on a registry database, the confounding bias cannot be ignored even with PSM. Thirdly, the relatively small sample size of the low-dose group might have impacted the statistical results. Fourthly, our study lacked mortality and functional outcomes in the long-term follow-up. Considering these limitations, further well-designed studies for patients with specialized characteristics are needed to provide more reference to medical practice.

## 5. Conclusions

Our observational study indicated that Chinese AIS patients receiving low-dose alteplase might be associated with an unfavorable functional outcome without lowering the risk of sICH. Our findings from the real-world dataset might provide more evidence to support standard-dose alteplase regimen for AIS patients in clinical practice.

## Data availability statement

The raw data supporting the conclusions of this article will be made available by the authors, without undue reservation.

## Ethics statement

This study was approved by the Institutional Review Board in Huashan Hospital, with the waiver of consent as no identifying information was collected.

## Author contributions

JX, XC, YX, YW, and SC performed the analysis. JX, XC, and YX drafted the manuscript. QD, YD, and KF revised the manuscript. YD and KF concepted this study, supervised the analysis, and finalized the manuscript. All authors contributed to the article and approved the submitted version.
