# Supplementary material for: Low-dose vs. standard-dose alteplase for Chinese patients with acute ischemic stroke: A propensity score analysis
Source: Front Neurol. 2023 Feb 21;14:1120547. doi: 10.3389/fneur.2023.1120547 (PMC9989159; doi:10.3389/fneur.2023.1120547)
Supplement: Supplementary file 1 [file Table_1.docx]

Supplementary Material

Low-dose vs. Standard-dose Alteplase for Chinese Patients with Acute Ischemic Stroke: A propensity score analysis

Jiawen Xu^1†^, Xi Chen^1†^, Yanan Xie^1†^, Yi Wang^1^, Shidong Chen^1^, Qiang Dong^1^, Yi Dong^1*^, Kun Fang^1*^

*** Correspondence:** Kun Fang & Yi Dong, Kun Fang: fangkun@huashan.org.cn. Yi Dong: drdongyi@yeah.net

# Supplementary Figures and Tables

## Supplementary Tables

**Supplementary Table 1. Baseline Characteristics of the Patients.**

|  | Standard-dose group,No. (%) | Low-dose group,No. (%) | P value |
| --- | --- | --- | --- |
| Patients, No. | 2740 | 439 |  |
| Age, y, mean (SD) | 67.2(11.6) | 73.2(14.2) | <0.001 |
| Men | 1775(64.8) | 268(61.0) | 0.13 |
| Medical history |  |  |  |
| Stroke | 402(14.7) | 97(22.1) | <0.001 |
| Diabetes | 660(24.1) | 97(22.1) | 0.36 |
| Hypertension | 1742(63.6) | 261(59.5) | 0.10 |
| Dyslipidemia | 89(3.3) | 13(3.0) | 0.75 |
| Myocardial infarction | 40(1.5) | 7(1.6) | 0.83 |
| Atrial fibrillation | 263(9.6) | 56(12.8) | 0.04 |
| Smoking | 937(34.2) | 126(28.7) | 0.02 |
| TOAST classification |  |  | <0.001 |
| Large-artery atherosclerosis | 1215(49.0) | 223(54.4) |  |
| Cardioembolism | 341(13.7) | 70(17.1) |  |
| Small-vessel occlusion | 756(30.5) | 74(18.0) |  |
| Other/unknown | 169(6.8) | 43(10.5) |  |
| Premorbid mRS score≤3 | 2512(93.0) | 387(89.8) | 0.02 |
| NIHSS, median (IQR) | 4(2,8) | 4(2,10) | 0.60 |
| LDL, mg/dL, median (IQR) | 2.8(2.3,3.5) | 2.8(2.2,3.4) | 0.19 |
| Time from stroke onset to intravenous thrombolysis, hr, median (IQR) | 2.6(1.8,3.4) | 2.6(1.9,3.4) | 0.23 |

Abbreviations: rtPA, recombinant tissue-type plasminogen activator; IQR, interquartile range; SD, standard deviation; CI, confidential interval; LDL, low-density lipoprotein; NIHSS, National Institutes of Health Stroke Scale (range, 0-42 [most severe]); mRS, modified Rankin scale (range, 0-6 [most severe]).

**Supplementary Table 2. The absolute standardized differences after PSM**

| Variable | Unmatched | Mean | |  | %reduct | t-test | | V(T)/V(C) |
| --- | --- | --- | --- | --- | --- | --- | --- | --- |
|  | Matched | Treated | Control | %bias | \|bias\| | t | p>\|t\| |  |
| Age | U | 73.898 | 67.408 | 50.3 |  | 9.67 | 0 | 1.48* |
|  | M | 73.782 | 73.138 | 5 | 90.1 | 0.66 | 0.508 | 1.27* |
| sex | U | 1.4037 | 1.3492 | 11.3 |  | 2.04 | 0.042 | 1.06 |
|  | M | 1.4032 | 1.4012 | 0.4 | 96.3 | 0.06 | 0.955 | 1 |
| Medical history | |  |  |  |  |  |  |  |
| Diabetes | U | 1.7754 | 1.7593 | 3.8 |  | 0.68 | 0.499 | 0.95 |
|  | M | 1.7742 | 1.7834 | -2.2 | 43.1 | -0.3 | 0.763 | 1.03 |
| Hypertension | U | 1.4037 | 1.3657 | 7.8 |  | 1.41 | 0.159 | 1.04 |
|  | M | 1.4005 | 1.4164 | -3.3 | 58.2 | -0.44 | 0.66 | 0.99 |
| Dyslipidemia | U | 1.9733 | 1.9712 | 1.3 |  | 0.22 | 0.822 | 0.93 |
|  | M | 1.9731 | 1.9677 | 3.3 | -156.6 | 0.43 | 0.666 | 0.84 |
| Myocardial infarction | U | 1.9893 | 1.9863 | 2.8 |  | 0.47 | 0.636 | 0.78* |
|  | M | 1.9892 | 1.9886 | 0.6 | 77.9 | 0.09 | 0.93 | 0.94 |
| Atrial fibrillation | U | 1.8824 | 1.9039 | -7 |  | -1.29 | 0.198 | 1.2 |
|  | M | 1.8817 | 1.8844 | -0.9 | 87.5 | -0.11 | 0.909 | 1.02 |
| Stroke | U | 1.7781 | 1.8535 | -19.5 |  | -3.71 | 0 | 1.38* |
|  | M | 1.7769 | 1.7444 | 8.4 | 57 | 1.04 | 0.3 | 0.91 |
| Smoking | U | 1.7246 | 1.6563 | 14.8 |  | 2.59 | 0.01 | 0.89 |
|  | M | 1.7258 | 1.7211 | 1 | 93.1 | 0.14 | 0.886 | 0.99 |
| Time from onset to treatment | U | 2.7539 | 2.6624 | 8.1 |  | 1.5 | 0.135 | 1.21 |
|  | M | 2.7552 | 2.7816 | -2.3 | 71.1 | -0.32 | 0.753 | 1.17 |
| Premorbid mRS | U | 1.639 | 1.4471 | 16.7 |  | 3.14 | 0.002 | 1.32* |
|  | M | 1.621 | 1.7386 | -10.2 | 38.7 | -1.28 | 0.2 | 0.86 |
| baseline NIHSS | U | 6.7701 | 6.0485 | 11.8 |  | 2.27 | 0.024 | 1.44* |
|  | M | 6.7608 | 6.9854 | -3.7 | 68.9 | -0.48 | 0.635 | 1.12 |
| TOAST classification | U | 1.8262 | 1.9982 | -15.4 |  | -2.71 | 0.007 | 0.9 |
|  | M | 1.8306 | 1.817 | 1.2 | 92.1 | 0.17 | 0.864 | 1 |
| LDL-C | U | 2.9713 | 2.9579 | 1 |  | 0.2 | 0.844 | 1.40* |
|  | M | 2.9789 | 2.9623 | 1.3 | -23.5 | 0.16 | 0.871 | 1.03 |

**Supplementary table 3. Outcome according to assigned treatment in the propensity score matching (1:1) dataset.**

| Outcome | Standard-dose group,  No. (%) | Low-dose  group,  No. (%) | Unadjusted Odds Ratio (95% CI) | P  value | Adjusted Odds Ratio (95% CI) * | P value |
| --- | --- | --- | --- | --- | --- | --- |
| Primary |  |  |  |  |  |  |
| mRS 2-6 | 126/310(40.7) | 156/323(48.3) | 1.36(1.00,1.92) | 0.05 | 1.59(1.10,2.29) | 0.01 |
| Secondary |  |  |  |  |  |  |
| sICH | 8/310(3.1) | 7/323(2.2) | 0.84(0.30,2.33) | 0.73 | 0.75(0.25,2.25) | 0.60 |
| In-hospital mortality | 3/310(1.0) | 4/323(1.2) | 1.28(0.28,5.78) | 0.75 | 1.32(0.26,6.62) | 0.74 |
| mRS 0-2 | 227/310(73.2) | 214/323(66.3) | 0.72(0.51,1.00) | 0.05 | 0.63(0.42,0.95) | 0.03 |
| mRS 0-3 | 247/310(79.7) | 249/323(77.1) | 0.86(0.59,1.25) | 0.43 | 0.83(0.53,1.30) | 0.42 |
| mRS, median (IQR) | 1(0,3) | 1(1,3) | 1.21(0.92,1.60) | 0.17 | 1.26(0.95,1.69) | 0.10 |
